# A method to assess linear self-predictability of physiologic processes in the frequency domain: application to beat-to-beat variability of arterial compliance

**DOI:** 10.3389/fnetp.2024.1346424

**Published:** 2024-04-04

**Authors:** Laura Sparacino, Yuri Antonacci, Chiara Barà, Dávid Švec, Michal Javorka, Luca Faes

**Affiliations:** ^1^ Department of Engineering, University of Palermo, Palermo, Italy; ^2^ Department of Physiology, Jessenius Faculty of Medicine in Martin, Comenius University in Bratislava, Martin, Slovakia

**Keywords:** linear autoregressive process, self-predictability, spectral decomposition, information theory, arterial compliance

## Abstract

The concept of self-predictability plays a key role for the analysis of the self-driven dynamics of physiological processes displaying richness of oscillatory rhythms. While time domain measures of self-predictability, as well as time-varying and local extensions, have already been proposed and largely applied in different contexts, they still lack a clear spectral description, which would be significantly useful for the interpretation of the frequency-specific content of the investigated processes. Herein, we propose a novel approach to characterize the linear self-predictability (LSP) of Gaussian processes in the frequency domain. The LSP spectral functions are related to the peaks of the power spectral density (PSD) of the investigated process, which is represented as the sum of different oscillatory components with specific frequency through the method of spectral decomposition. Remarkably, each of the LSP profiles is linked to a specific oscillation of the process, and it returns frequency-specific measures when integrated along spectral bands of physiological interest, as well as a time domain self-predictability measure with a clear meaning in the field of information theory, corresponding to the well-known information storage, when integrated along the whole frequency axis. The proposed measure is first illustrated in a theoretical simulation, showing that it clearly reflects the degree and frequency-specific location of predictability patterns of the analyzed process in both time and frequency domains. Then, it is applied to beat-to-beat time series of arterial compliance obtained in young healthy subjects. The results evidence that the spectral decomposition strategy applied to both the PSD and the spectral LSP of compliance identifies physiological responses to postural stress of low and high frequency oscillations of the process which cannot be traced in the time domain only, highlighting the importance of computing frequency-specific measures of self-predictability in any oscillatory physiologic process.

## 1 Introduction

In the past decades, zealous interdisciplinary research studies have been focused on the spontaneous oscillations exhibited by variables describing the temporal dynamic activity of several physiologic systems. These oscillatory patterns can arise from the combined action of multiple regulatory mechanisms, including mechanical coupling effects and autonomic nervous system control, often arranged in closed-loop interactions among interconnected subsystems reacting to internal and external stimuli (Glass, 2001). Since these interactions occur across a wide range of time scales, e.g., spanning from 0.02 to 0.5 Hz in the case of cardiovascular and respiratory systems, the time course of the investigated physiologic variables is characterized by different degrees of regularity (i.e., recurrence of specific patterns with different dominant frequencies and shapes). A variety of techniques has been proposed to quantify the richness of a dynamic process, as well as to characterize the system state in various physiological and pathological conditions (Pincus 1994; Porta et al., 1998; Richman and Moorman 2000; Goldberger et al., 2002; Porta et al., 2007; Porta et al., 2012; Faes et al., 2015b; Valente et al., 2018). A commonly used approach is the quantification of the degree of regularity, or *self-predictability*, of patterns extracted from short-term recordings intended as realizations of dynamic processes. To this aim, different univariate measures have been proposed throughout the years, either derived from nonlinear prediction (Porta et al., 2006; Erla et al., 2011) or based on the concepts of conditional entropy and information storage (Porta et al., 1998; Richman and Moorman, 2000; Lizier et al., 2012; Faes et al., 2013; Wibral et al., 2014). Conditional entropy has been referred to as a measure of complexity quantifying the rate of entropy generation of an individual system, while the notion of information storage, representing information from the past of a stochastic process to predict its future (Lizier et al., 2012; Wibral et al., 2014), has allowed quantification of self-dynamics. This has been achieved through the well-established measure of self-entropy (Barnett et al., 2009; Faes et al. (2015b; 2016), serving as a gauge of self-predictability by quantifying the information shared between the current and the past states of the process.

A drawback of information-theoretic measures quantifying self-predictability is that they represent global indexes which address the entire temporal evolution of the analysed process, without obtaining time-resolved information about its dynamics nor focusing on specific frequency rhythms (Faes et al., 2015a). On the other hand, physiological time series display a rich oscillatory content, which is typically manifested within the low frequency (LF, 0.04 − 0.15 Hz) and high frequency (HF, 0.15 − 0.4 Hz) bands of the spectrum in the case of cardiovascular and respiratory variables (Cohen and Taylor, 2002; Shaffer et al., 2014). This limitation has already been addressed through the definition of time-varying (Valenza et al., 2017) and local (Faes et al., 2016; Barà et al., 2023) measures of information storage, the latter defining how information is stored at each moment in time in a stochastic process (Lizier et al. (2008; 2012); Lizier (2014)). A different approach has been proposed in (Donoghue et al., 2020), where the periodic and aperiodic oscillations of a process have been identified in the power spectrum, e.g., by exploiting the power spectral density obtained via parametric or non parametric methods. Despite this methodology owns a great potential in accurately quantifying the frequency-specific content of physiological variables, it does not take into account measures from the field of information theory.

In this work, we aim to fill the gap by introducing a novel methodology that combines the two frameworks, i.e., information-theoretic and frequency domain approaches. We propose a new approach for the linear spectral analysis of self-predictability in physiological processes, which allows to obtain frequency-resolved information about their dynamics (Sect. 2). A novel spectral measure of linear self-predictability (LSP) is derived intuitively from the transfer function (TF) of the autoregressive (AR) linear model describing the relationship between the current and past states of the process, and is defined in such a way to match the time domain information storage if integrated alongside the whole frequency axis (Geweke, 1982; Chicharro, 2011). The proposed measure is first illustrated in a theoretical example, where its behaviour at varying the radii of the poles of the AR TF is shown (Sect. 3). Moreover, we demonstrate that the dominant peaks in the spectrum of the simulated Gaussian process, which reflect regular (thus self-predictable) oscillations in the same process, can be easily detected by our method that indeed constitutes a bridge between the information-theoretic and the frequency domain frameworks. Furthermore, in order to display the physiological significance and potentiality of our approach, the LSP measure is computed over short-term beat-to-beat time series of arterial compliance (AC) measured in young healthy subjects using a recently developed noninvasive methodology (Švec et al., 2021) (Sect. 4). Being a cardiovascular variable characterizing mechanical and structural properties of the arteries (Švec and Javorka, 2021), very little is known about the short-term-variability nature of AC. Arterial compliance variability is expected to be affected by the sympathetic and vagal activities, whose interaction reflects the typical balance between fight-or-flight and rest-or-digest responses (McCorry, 2007; Ondicova and Mravec, 2010; Gibbons, 2019). Investigating the spectral content of AC, as well as understanding how this parameter behaves in response to external perturbations, is important to characterize its beat-to-beat dynamics for physiological research and clinical purposes (Mackenzie et al., 2002; Sugawara et al., 2012; Tan et al., 2016). Moreover, assessing the presence and extent of LF oscillations in the beat-to-beat variability of arterial compliance may be highly relevant for the exploration of sympathetic activity changes, assumed as the dominant LF-driven responsible for the dynamics of this parameter, except of mechanical effects (e.g., effects of intrathoracic pressure or arterial blood pressure). Therefore, since we are not aware of past studies performing a systematic spectral characterization of this physiological variable, here we aimed to assess, separately for LF and HF oscillations, its degree of self-predictability in the resting state and in response to the alterations in the sympatho-vagal balance induced by postural stress. We exploited the well-known method of spectral decomposition (Baselli et al., 1997; Pernice et al., 2021) to retrieve pole-specific LSP profiles, as well as the power content associated to specific oscillations with central frequency in LF and HF bands of the spectrum.

Overall, in spite of the complex mathematical framework mixing together concepts from the fields of information theory and spectral analysis of linear Gaussian processes, our results evidence (i) the feasibility of our approach and its worthwhile generalization to a variety of physiological processes displaying oscillatory activity, as well as (ii) the need of computing spectral measures of self-predictability focusing on specific frequency bands with physiological meaning, to reveal mechanisms which remain otherwise hidden in a whole-band time domain analysis.

## 2 Linear autoregressive models of physiologic processes

Let us consider the stochastic process *Y*, with *Y*_*n*_ denoting the random variable sampling the process at time *n*, where *n* is the temporal counter, and $Y_{n}^{-}=\left[Y_{n-1},Y_{n-2},\ldots \right]$ the random vector describing its past states. Broadly speaking, separating the present from the past states of a given process allows to consider the flow of time and to investigate the causal interactions within the process by looking at the statistical dependencies among the random variables describing those states. Importantly, the Markov property allows to investigate the state transitions relevant to the considered process by restricting the analysis to the past *p* states visited by the process itself, such that the present state depends on the past states only through a finite number of time steps, i.e., $Y_{n}^{p}=\left[Y_{n-1},Y_{n-2},\ldots ,Y_{n-p}\right]$ .

Assuming *Y* as a stationary Markov process, the following linear AR model can be used to study the dynamic interactions between its present and past states (Lütkepohl, 2005):

$$Y_{n}=\overset{p}{\underset{k=1}{\sum }}a_{k}Y_{n-k}+U_{n},$$ (1)

where *p* is the model order, describing the maximum lag used to quantify interactions, *U*_*n*_ is the present state of a scalar zero-mean white process with variance $\sigma_{U}^{2}$ , *a*_*k*_ is the coefficient describing the interaction from *Y*_*n*−*k*_ to *Y*_*n*_ at lag *k*.

### 2.1 Spectral decomposition of oscillatory content

The AR model in Eq. (1) can be represented in the Z-domain through its Z-transform yielding *Y*(*z*) = *H*(*z*)*U*(*z*), where $H\left(z\right)={\left[1-\sum_{k=1}^{p}a_{k}z^{-k}\right]}^{-1}=\bar{A}{\left(z\right)}^{-1}$ is the TF modelling the relationship between the input *U*(*z*) and the output *Y*(*z*). Applying the residue theorem, the latter can be expressed as (Baselli et al., 1997):

$$H\left(z\right)=\frac{z^{p}}{\prod_{k=1}^{p}\left(z-p_{k}\right)}=\overset{p}{\underset{k=1}{\prod }}H^{\left(k\right)}\left(z\right),$$ (2)

where *p*_*k*_, *k* = 1, …, *p*, are the *p* poles of the AR process, i.e., the roots of $\bar{A}\left(z\right)$ , while the terms $H^{\left(k\right)}\left(z\right)=\frac{z}{z-p_{k}}\cdot \frac{1/z^{*}}{1/z^{*}-p_{k}}$ are pole-specific factors associated each to a given pole *p*_*k*_, with * the Hermitian transpose. Then, the power spectral density (PSD) of the process can be written in the Z-domain as $P\left(z\right)=H\left(z\right)\sigma_{U}^{2}H^{*}\left(\frac{1}{z^{*}}\right)$ , and expanded exploiting the Heaviside decomposition with simple fractions relevant to all the poles and weighted by the relevant residuals of *P*(*z*), to get (Baselli et al., 1997; Pernice et al., 2021):

$$P\left(z\right)=\overset{Q}{\underset{q=1}{\sum }}P^{\left(q\right)}\left(z\right)=\overset{Q}{\underset{q=1}{\sum }}\left[\frac{r_{q}p_{q}}{z-p_{q}}-\frac{r_{q}p_{q}^{-1}}{z-p_{q}^{-1}}\right],$$ (3)

where $r_{q}=\frac{\sigma_{U}^{2}}{z\prod_{h\neq q}\left(z-p_{h}\right)\cdot \prod \left(z^{-1}-p_{h}\right)}{|}_{z=p_{q}}$ are the residuals of *P*(*z*), *q* = 1, …, *Q*. Note that the number of components of the PSD is *Q* ≃ *p*/2 depending on the number of real poles; specifically, there is one component for each real pole and for each pair of complex conjugate poles. Then, by computing *P*(*z*) on the unit circle of the complex plane, $P\left(f\right)=P\left(z\right){|}_{z=e^{j2\pi f/f_{s}}}$ , where *f* ∈ [0, *f*_*s*_/2], with *f*_*s*_ the sampling frequency, it is possible to obtain the spectral profile of the process, *P*(*f*), as well as of the *q*th component, *P* ^(*q*)^(*f*). Crucially, each spectral component *P* ^(*q*)^(*f*) is described by a specific profile that is shaped by the corresponding TF factor $|H^{\left(q\right)}\left(f\right){|}^{2}=H^{\left(q\right)}\left(z\right)H^{*\left(q\right)}\left(z\right){|}_{z=e^{j2\pi f/f_{s}}}$ , and has an associated frequency (related to the pole frequency, $f_{q}=\frac{arg\left\{p_{q}\right\}}{2\pi }$ ) and power (related to the pole residual, $\sigma_{q}^{2}=r_{q}$ for real poles and $\sigma_{q}^{2}=2\cdot R\left\{r_{q}\right\}$ for complex conjugate poles). Note that the sum of the pole variances $\sigma_{q}^{2}$ , with *q* = 1, …, *Q*, equals the total power of the process, which represents its variance $\sigma_{Y}^{2}$ .

Importantly, in comparison to classical approaches based on integrating the PSD profile within the spectral bands of interest to get band-specific time domain powers (Krohova et al., 2020), the proposed method of spectral decomposition allows to focus only on the spectral components with frequencies within those bands, thus avoiding spurious contributions due to broadband oscillations. This peculiarity is exploited in the next subsection to define a pole-specific measure of self-predictability in the frequency domain.

### 2.2 Spectral decomposition of self-predictability

In the previous section we have seen how analysing the spectrum of a physiologic process provides noteworthy information on the frequency-specific location of the oscillations of that process, thus allowing to identify and separate its different spectral components. Here, we show how the characterization of the spectral dynamics of a process can be also carried on by looking at its degree of linear self-predictability, drawing a connection with information theory.

The most popular information-theoretic measure of self-predictability is the information storage (IS), which quantifies, for a random process *Y*, the amount of information contained in the present state *Y*_*n*_ that can be predicted by the knowledge of its past states, $Y_{n}^{-}$ (Lizier et al., 2012). In the linear signal processing framework, the IS has a straightforward formulation that involves the variance of the process and the variance of the prediction error of its AR representation (Barnett et al., 2009; Faes et al., 2015b):

$$S_{Y}=\frac{1}{2}\ln\left(\frac{\sigma_{Y}^{2}}{\sigma_{U}^{2}}\right),$$ (4)

where $\sigma_{Y}^{2}$ is the variance of *Y* and $\sigma_{U}^{2}$ is the variance of the residual in Eq. (1), i.e., the partial variance of *Y*_*n*_ given its past $Y_{n}^{p}$ . The quantity defined in Eq. (4) is a measure of LSP in the time domain. To expand it in the frequency domain, we exploit the spectral representation of the AR model in Eq. (1) and the spectral decomposition described in the previous and represented by Eqs. (2, 3). We start noting that the TF *H*(*z*) contains spectral information about the predictable dynamics of *Y*, as it is directly derived from the Z-domain representation of the AR model coefficients *a*_*k*_, *k* = 1, …, *p*, which in turn describe these dynamics in the time domain. Then, we exploit the fact that such frequency-specific information can be particularized to each oscillatory component considering the TF factor |*H* ^(*q*)^(*f*)|^2^, so as to retrieve information on the pole-specific self-dynamics of the AR process. This factor is the squared TF associated to a real pole or the squared product of the two TFs associated to a pair of complex conjugated poles. We expect that stronger self-dynamics of *Y* at the frequency *f*_*q*_ are reflected by higher values of |*H* ^(*q*)^(*f*)|^2^, which indeed shows a positive peak at that frequency. Therefore, we define the frequency-specific spectral LSP measure as

$$s_{Y}^{\left(q\right)}\left(f\right)=\frac{1}{2}\ln\left(\frac{\sigma_{Y}^{2}|H^{\left(q\right)}\left(f\right){|}^{2}}{\sigma_{U}^{2}}\right).$$ (5)

The spectral in Eq. (5) can be written also as $s_{Y}^{\left(q\right)}\left(f\right)=\frac{1}{2}\ln\left(\frac{\sigma_{Y}^{2}}{\sigma_{U}^{2}}\right)+\frac{1}{2}\ln\left(|H^{\left(q\right)}\left(f\right){|}^{2}\right)=S_{Y}+{\bar{s}}_{Y}^{\left(q\right)}\left(f\right)$ , and satisfies the spectral integration property (Geweke, 1982), i.e., it is such that its integral extended over all frequencies returns the time-domain LSP measure:

$$S_{Y}=\frac{1}{2\pi }\int_{-\pi }^{\pi }s_{Y}^{\left(q\right)}\left(f\right)df.$$ (6)

Note that Eq. (6) is satisfied because the full-frequency integral of the term ${\bar{s}}_{Y}^{\left(q\right)}\left(f\right)$ is null, i.e., $\int_{-\pi }^{\pi }\ln\left(|H^{\left(q\right)}\left(f\right){|}^{2}\right)df=0$ (Rozanov 1967; Chicharro, 2011). Therefore, the spectral LSP consists of a frequency-independent part equal to *S*_*Y*_ and a frequency-specific part quantified by ${\bar{s}}_{Y}^{\left(q\right)}\left(f\right)$ , which takes negative values at some frequencies, depending on where the informative content is located.

The spectral decomposition of the pole-specific LSP into terms related to the *Q* oscillations of the AR process, depicted in Eq. (5), allows to locate the self-dynamics of *Y* in specific spectral bands with given frequency, as well as to compute their strength as the integral of these profiles within the investigated bands. Remarkably, the spectral LSP profiles display peaks as the PSD does, since both are derived from adaptations of the TF of the AR model describing the data. The difference between the two resides in the logarithmic formulation of the LSP in the well-known framework of information theory. Indeed, being a measure of information shared between the present and past states of the investigated processes according to its mathematical definition, it can be quantified in natural units (*nats*) in the time domain, and in *nats/Hz* in the spectral domain, thus acquiring a clear meaning in the context of information theory.

### 2.3 Statistical validation

This section presents the use of surrogate and bootstrap data analyses to assess the statistical significance of the proposed measures of time domain and spectral LSP.

As far as we know, the task of validating the presence of a significant non-flat component within the spectrum of a process is not straightforward. In the spirit of surrogate data analysis, one should destroy the oscillation under scrutiny without altering the remaining spectral patterns of the process. However, this is undoubtedly challenging and requires in-depth analyses. Therefore, in this work we propose an empirical approach based on bootstrap data analysis (Politis, 2003) to assess the statistical significance of pole-specific measures of self-predictability, while time domain measures are validated through the well-known method of surrogate data analysis (Theiler et al., 1992). Validation is performed at the level of individual realizations of the observed process *Y*, obtained in the form of the time series **y** = {*y*(1), …, *y*(*M*)}, where *M* is the length of the time series. Specifically, a linear model as in Eq. (1) is first identified on the time series **y** through the vector least square approach; then, estimates of the spectral and the time domain LSP measures, denoted respectively as $s_{y}^{\left(q\right)}\left(f\right)$ , with *q* = 1, …, *Q*, and *S*_**y**_, are obtained from the estimated model parameters using Eqs. (5, 6). The spectral profiles $s_{y}^{\left(q\right)}\left(f\right)$ are integrated within the spectral band of interest *F* to get estimates of self-predictability in that band, indicated as $s_{y}^{\left(F\right)}$ .

#### 2.3.1 Surrogate data analysis

The method of *surrogate data* (Theiler et al., 1992) is employed to obtain a threshold for zero self-predictability setting a significance level for the time domain LSP measure. Specifically, randomly shuffled surrogates (Palus, 1997), which are realizations of independent and identically distributed (i.i.d.) stochastic processes with the same mean, variance and probability distribution as the original series, are generated by randomly permuting in temporal order the samples of the original series, according to the null hypothesis of absence of autonomous dynamics within the investigated process. This procedure is repeated *N*_*s*_ times to obtain the surrogate series **y** ^*s*^, *s* = 1, …, *N*_*s*_. The time domain LSP is then estimated on each surrogate, yielding the distribution $S_{y}^{s}$ , from which the significance threshold $S_{y}^{\alpha }$ is derived taking the 100(1 − *α*)^*th*^ percentile. Finally, the original time domain LSP value is deemed as statistically significant if $S_{y}>S_{y}^{\alpha }$ . In this work, *N*_*s*_ = 100 surrogate pairs were generated to assess the existence of significant self-predictability in the time domain.

#### 2.3.2 Bootstrap data analysis

The *block bootstrap* data generation procedure (Politis, 2003) is followed to generate, starting from the time series **y**, *N*_*b*_ bootstrap pseudo-series **y** ^*b*^ = {*y* ^*b*^(1), …, *y* ^*b*^(*M*)}, *b* = 1, …, *N*_*b*_, which maintain all the individual properties of the original time series, i.e., mean, variance and probability distribution. The bootstrap pseudo-series are generated by feeding the AR model identified on the original time series **y** with bootstrap pseudo-residuals. The procedure creates the bootstrap pseudo-residuals **u** ^*b*^ = {*u* ^*b*^(1), …, *u* ^*b*^(*M*)} by joining together $l=\frac{M}{L}$ non-overlapping blocks chosen randomly from the set {*B*_1_, *…*, *B*_*l*_}, where *L* is the size of each block, *B*_*m*_ = {**u**(*m*), *…*, **u**(*m* + *L* − 1)} and *m* is chosen randomly from the set {1, *…*, *M* − *L* + 1}. After generation of the bootstrap time series **y** ^*b*^ from the original AR model coefficients *a*_*k*_, *k* = 1, …, *p*, and the bootstrap pseudo-residuals **u** ^*b*^, the time domain and spectral LSP profiles are recomputed from the new, full-size bootstrap series **y** ^*b*^ to get the estimates $S_{y}^{b}$ and $s_{y}^{{\left(q\right)}^{b}}\left(f\right)$ , respectively, with *q* = 1, …, *Q*; the spectral profiles are then integrated in the desired band *F* to get the estimates $s_{y}^{{\left(F\right)}^{b}}$ . The procedure is iterated for *b* = 1, …, *N*_*b*_ to construct bootstrap distributions. The significance threshold $s_{y}^{{\left(F\right)}^{\alpha }}$ is derived taking the *α* ^*th*^ percentile of the distribution. In order to assess the existence of significant self-predictability in *F*, we exploit the fact that the spectral profile $s_{y}^{{\left(F\right)}^{b}}\left(f\right)$ oscillates around the value *S*_**y**_, which does not vary with *f*. Then, the core of the procedure lies in evaluating the degree of emergence of the peak of $s_{y}^{{\left(F\right)}^{b}}\left(f\right)$ in *F* with respect to the mean value assumed by the same spectral profile if the oscillation is not present, i.e., *S*_**y**_. To this purpose, the original pole-specific spectral LSP value integrated in *F*, $s_{y}^{\left(F\right)}$ , is deemed as statistically significant if $s_{y}^{{\left(F\right)}^{\alpha }}>\frac{1}{\Delta F}\int_{F}S_{y}\left(f\right)df$ , where Δ*F* is the bandwidth and *S*_**y**_(*f*) is the spectral profile of the original time domain LSP, equal to *S*_**y**_ *∀f*. In this work, *N*_*b*_ = 100 bootstrap repetitions were generated to identify confidence intervals for the investigated measures.

## 3 Validation on simulations

In this section, we study the behavior of the proposed self-predictability measure using a simulated AR process, where the exact profiles of the spectral LSP are computed (with sampling frequency *f*_*s*_ = 1 Hz) from the true values imposed for the AR parameters. The process *Y*, exhibiting autonomous oscillations at different frequencies, is defined as:

$$Y_{n}=\overset{4}{\underset{k=1}{\sum }}a_{k}Y_{n-k}+U_{n}$$ (7)

where *U* is a Gaussian white noise process with zero mean and unit variance. The autonomous oscillations of *Y* are obtained placing pairs of complex-conjugate poles, with modulus *ρ* and phase 2*πf*, in the complex plane representation of the process; the AR coefficients resulting from this setting are *a*_1_ = 2*ρ* cos(2*πf*) and *a*_2_ = −*ρ* ^2^ (Faes et al., 2015b). Here, we imposed autonomous oscillations for the process *Y* in the LF and HF bands of the spectrum, setting *ρ*_*HF*_ = 0.9, *f*_*HF*_ = 0.3 Hz, so that the dynamics of *Y* in the HF band are determined by the fixed coefficients *a*_1_ = −0.556, *a*_2_ = −− 0.81, and *ρ*_*LF*_ = *b* ⋅ 0.8, *f*_*LF*_ = 0.1 Hz, so that the strength of the dynamics of *Y* in the LF band, which are determined by the coefficients *a*_3_, *a*_4_, depends on the parameter *b* varying in the range $\left[0,1\right]$ . The theoretical values of the time domain and the spectral LSP measures are computed for each value of the parameter *b*.

Furthermore, *N*_*b*_ = 100 bootstrap realizations of the process *Y*, each of length *N* = 1000 points, are then generated *∀b* by feeding (7) with *N*_*b*_ block bootstrap versions of a single realization of the white noise process *U*, using the theoretical coefficients *a*_*k*_, *k* = 1, …, 4. The time domain and spectral LSP measures are then estimated after identifying the AR model fitting the bootstrap time series of *Y*; the model order was set using the Akaike Information Criterion (AIC). Statistical significance of the spectral LSP measures in a given frequency band is then assessed exploiting the method described in Sect. 2.3.2.

**FIGURE 1:**
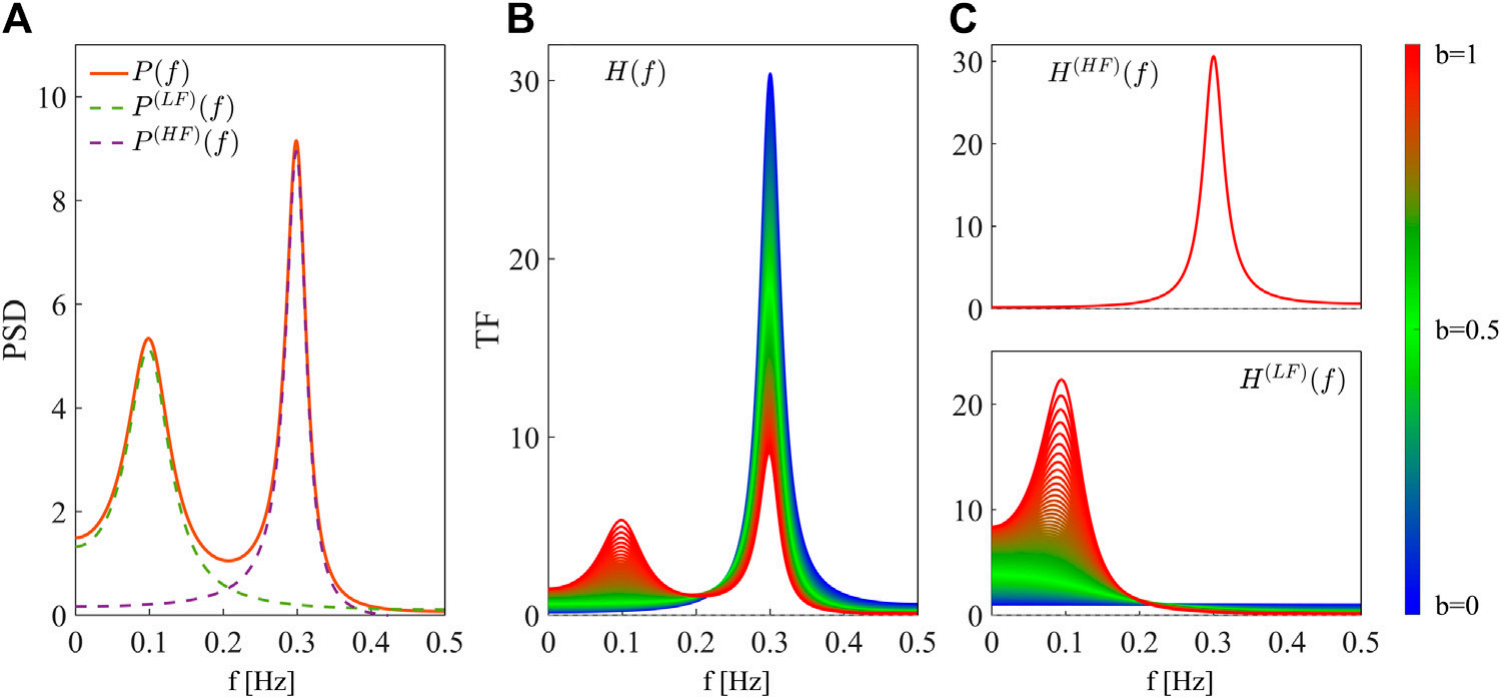
Spectral decomposition of the power spectral density and the transfer function of the simulated AR process. **(A)** Linear spectral decomposition of the process *Y* with *b* = 1. The PSD, *P* **(f)** (orange continuous line), is decomposed into components *P* ^(LF)^(*f*) (green dashed line) and *P* ^(HF)^(*f*) (purple dashed line) with associated frequency *f*_LF_, *f*_HF_ and power $\sigma_{\text{LF}}^{2}$ , $\sigma_{\text{HF}}^{2}$ . **(B)** Spectral profiles of the TF of the process, *H* **(f)**. **(C)** Spectral profiles of the frequency-specific TFs of the process, computed for the poles with frequency inside the HF ([0.15 − 0.4]*Hz*, *H* ^(*HF*)^(*f*), above) and LF ([0.04 − 0.15]*Hz*, *H* ^(*LF*)^(*f*), below) bands of the spectrum. Profiles are computed at varying the parameter *b* from 0 (blue continuous line) to 1 (red continuous line).

The spectral decompositions of the PSD and the TF of the simulated AR process are reported in Figure 1. Figure 1A shows the theoretical PSD profile (orange continuous line) of the process *Y* when *b* = 1, decomposed into its two spectral components, LF (green dashed line) and HF (purple dashed line). Figure 1B shows the theoretical profiles of the TF of the AR process, *H*(*f*), at varying the parameter *b* from 0 (blue) to 1 (red). The TF shows only a positive peak in the HF band in absence of LF dynamics, i.e., when *b* = 0, and a positive peak in LF with amplitude increasing with the parameter *b*. This demonstrates that the TF is sensitive to the oscillatory content of the AR process, and it peaks wherever its self-dynamics are located in the frequency domain. Indeed, the two TF contributions in HF (Figure 1C, above) and LF (Figure 1C, below) display frequency-specific peaks with constant or varying amplitude depending on how the corresponding dynamics are modulated.

**FIGURE 2:**
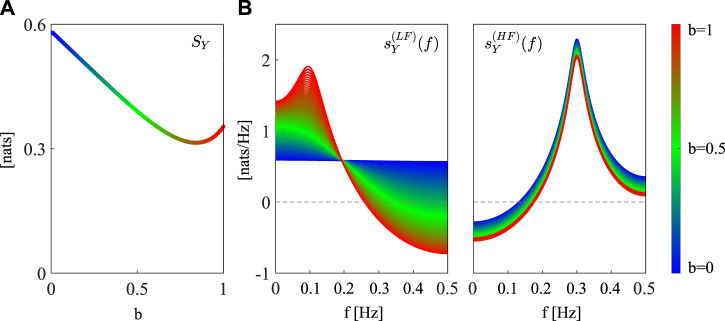
Spectral decomposition of the linear self-predictability of the simulated AR process. **(A)** Profile of *S*_*Y*_ at varying the parameter *b* from 0 (blue dots) to 1 (red dots). **(B)** Spectral profiles of the frequency-specific terms in Eq. (5) computed for the poles with frequency inside the LF ([0.04 − 0.15]*Hz*, $s_{Y}^{\left(LF\right)}\left(f\right)$ , left) and HF ([0.15 − 0.4]*Hz*, $s_{Y}^{\left(HF\right)}\left(f\right)$ , right) bands of the spectrum. Spectral profiles are computed at varying the parameter *b* from 0 (blue continuous line) to 1 (red continuous line).

The theoretical spectral profiles of the LSP measures resulting from the simulation are reported in Figure 2. The time domain LSP *S*_*Y*_ exhibits a non-monotonic behavior at varying this parameter from 0 to 1 (blue to red dots, Figure 2A). Specifically, the increase of the parameter *b* determines an initial decrease of LSP, followed by a slight increase when *b* = 1. Thus, high regularity is found whenever the process has only one oscillation, while the presence of two oscillations makes the process less predictable. Only when *b* approaches 1 (Figure 2B, left, reddish profiles), the emergence of a clear LF oscillation determines a decrease of $\sigma_{U}^{2}$ and thus an increase of *S*_*Y*_ (Figure 2A, reddish dots). The frequency-specific terms show positive peaks at the LF and HF frequencies (0.1 Hz and 0.3 Hz, respectively), confirming that the system owns self-dynamics and is thus self-predictable at the frequencies of the PSD peaks ( $s_{Y}^{\left(q\right)}\left(f\right)$ , where *q* indicates the LF or HF band, Figure 2B). Specifically, while the HF contribution (right) does not change consistently with *b* and is always significant according to bootstrap data analysis, the LF profile (left) is constant over frequencies and equal to *S*_*Y*_ when *b* = 0, while a peak in the LF band emerges gradually with increasing values of *b*. Only when *b* > 0.6, the bootstrap procedure provided significant results for the LF oscillation, thus allowing to statistically assess the existence of significant self-predictability in this band.

To sum up, in the proposed simulated example we showed that the spectral LSP is able to quantify and localize the self-dynamics of *Y* in the frequency domain, thus reflecting their presence and strength and showing positive peaks at the frequencies where they are located. Its time domain counterpart rather provides an overall description of these dynamics without focusing on specific oscillatory rhythms. Remarkably, the utilization of surrogate and bootstrap data analyses allows to assess the statistical significance of the proposed measures in the time and frequency domain, thus enabling to get more confidence in the significance of the findings and draw meaningful conclusions.

## 4 Application to arterial compliance short-term variability analysis

### 4.1 Study protocol, data acquisition and time series extraction

This section reports the application of the described methods on arterial compliance time series taken from a larger database recently collected (Švec et al., 2021). The original study, approved by the ethical committee of the Jessenius Faculty of Medicine, Comenius University, included a total of 81 young and healthy Caucasians, aged 18.56 ± 2.88 years.

Arterial blood pressure (ABP) signal from finger, obtained by the photoplethysmographic volume-clamp method followed by brachial ABP reconstruction (Finometer Pro, FMS Netherlands), and electrocardiogram (ECG, CardioFax ECG-9620, NihonKohden Japan) were recorded during two phases of the experimental protocol: (i) the resting supine position (REST), started 8 min after the beginning of the measurement, and (ii) the upright position reached after passive head-up tilt (TILT), started 3 min after the position change from supine to tilt. Heart period (HP) intervals were extracted as the time distance between consecutive R peaks of the ECG. Hemodynamics parameters including cardiac output (CO) were derived on a beat-to-beat basis exploiting the impedance cardiography (ICG, CardioScreen 2000; Medis, Germany) and exerted a main role in the subsequent determination of the AC time series. The value of AC was quantified through a recently developed method, based on a reliable estimation of the time constant *τ*, i.e., the rate of the peripheral ABP decay during the diastolic phase, for each heart beat separately, as well as on the exploitation of the relationship between *τ*, AC and the total peripheral resistance (TPR) based on the two-element Windkessel model (Švec and Javorka, 2021). Since the measurement of hemodynamic parameters using ICG is very sensitive to movement artifacts, skin condition and distribution of fat, in some cases these parameters were then not determined for each heart beat, and then only 39 subjects were selected for further analysis. All the acquired signals were digitized at a sampling rate of 1 kHz. Transient changes in cardiovascular parameters between consecutive phases of the study protocol were excluded from analysis. Then, stationary segments of 300 consecutive beats were extracted from the original recordings in the two phases of the protocol. We refer the reader to (Švec et al., 2021) for further details about data acquisition and time series extraction.

### 4.2 Data analysis and statistical validation

The time series extracted for each subject in the two experimental conditions were regarded as realizations of the AC discrete-time process (in the following, referred to as *C*), assumed as uniformly sampled with a sampling frequency equal to the inverse of the mean HP. First, classical time domain markers, i.e., the mean and variance of AC ( $\mu_{C}\left[\frac{mL}{mmHg}\right]$ and $\sigma_{C}^{2}\left[\frac{mL^{2}}{mmHg^{2}}\right]$ ) were computed. Then, the series were pre-processed by removing the mean value. The AR model (1) was fitted on each pre-processed series using vector least-squares identification and setting the model order *p* according to the Akaike Information Criterion (AIC) (maximum scanned model order equal to 14). Since the use of the AIC sometimes led to duplicate peaks or negative power as a result of spectral decomposition (Pernice et al., 2021), the model order was manually adjusted so as to detect spectral components with positive power. After AR identification, the spectral profiles were computed according to (3). Moreover, the LF and HF components, i.e., *P* ^(LF)^(*f*) and *P* ^(HF)^(*f*) respectively, were computed from the poles with central frequency located in the ranges $\left[0.04-0.15\right]$ Hz and $\left[0.15-0.4\right]$ Hz, respectively, and the related variance was obtained from the pole residuals ( $\sigma_{\text{LF}}^{2}$ and $\sigma_{\text{HF}}^{2}$ ). For some subjects, more than one peak was found in these bands; in such cases, the poles with the highest power were selected for further analysis. Finally, the spectral profiles of the LSP measure in (5), computed for the LF and HF oscillations, were integrated in these bands and marked as $s_{C}^{\left(LF\right)}$ , $s_{C}^{\left(HF\right)}$ , respectively. The time domain LSP was obtained exploiting (6) and marked as *S*_*C*_.

To test the statistical significance of the time and frequency domain LSP measures, surrogate and bootstrap data analyses were implemented as described in Sect. 2.3.1 and 2.3.2, respectively.

As regards statistical analysis, the distributions of the computed measures were tested for normality using the Anderson-Darling test. Since the hypothesis of normality was rejected for most of the distributions, and given the small sample size, non-parametric tests were employed. Specifically, the statistical significance of the difference between REST and TILT conditions, as well as between integrated PSD values in LF and HF bands in a given experimental condition, was assessed using the Wilcoxon signed-rank test for paired data. In this work, a significance level *α* = 0.05 was used to compute confidence intervals of the surrogate and bootstrap distributions as well as to conduct statistical tests.

### 4.3 Results and discussion

The results of the time domain analysis are reported in Table 1, revealing that both the mean *μ*_*C*_ and the variance $\sigma_{C}^{2}$ of the AC time series decreased significantly with head-up tilt (*p* < 0.001). This is in accordance with previous findings (Hasegawa and Rodbard, 1979; Huijben et al., 2012; Švec et al., 2021) and suggests that, when higher sympathetic activity is assumed, i.e., during the orthostatic challenge, the well-known changes of heart rate and total peripheral resistance occur rapidly through baroreflex mechanisms (Cooper and Hainsworth, 2001; Sugawara et al., 2012; Nardone et al., 2018), and are accompanied by a simultaneous rise in arterial stiffness.

**TABLE 1:** Time domain indexes (mean *μ*_*C*_ and variance $\sigma_{C}^{2}$ ) of AC in the REST and TILT experimental conditions. Values are computed over 39 subjects and expressed as mean ± standard deviation. Wilcoxon signed rank test for paired data:* *p* < 0.05 REST vs. TILT.

	REST	TILT
$\mu_{C}\left[\frac{mL}{mmHg}\right]$	1.76 ± 0.41	1.42 ± 0.28*
$\sigma_{C}^{2}\left[\frac{mL^{2}}{mmHg^{2}}\right]$	0.022 ± 0.015	0.015 ± 0.008*

**FIGURE 3:**
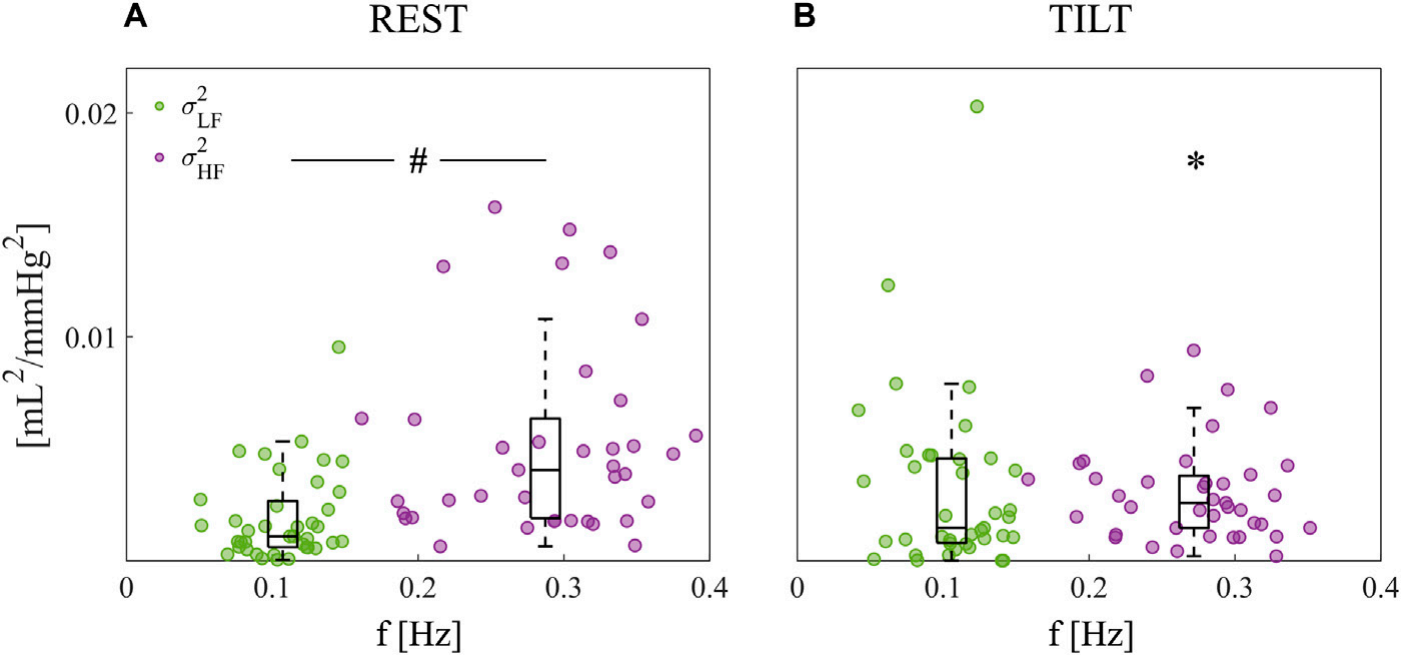
Spectral decomposition of the beat-to-beat arterial compliance time series. Power of AC computed at rest **(A)** and during tilt **(B)**; powers are depicted as boxplot distributions and individual values with coordinates $\left(f_{\text{LF}},\sigma_{\text{LF}}^{2}\right)$ (green circles) and $\left(f_{\text{HF}},\sigma_{\text{HF}}^{2}\right)$ (purple circles). The total number of subjects is 39, but only the subjects for those the algorithm detected at least one pole in the LF and HF bands are shown here. Statistically significant differences assessed by the Wilcoxon signed-rank test for paired data (∗, *p* < 0.05 REST vs. TILT; *#*, *p* < 0.05 LF vs. HF).

Figure 3 shows the boxplot distributions of the spectral power of AC in the REST (Figure 3A) and TILT (Figure 3B) conditions computed within the LF ( $\sigma_{LF}^{2}$ , green circles) and HF ( $\sigma_{HF}^{2}$ , purple circles) bands, and depicted in a way such that subject-specific information relevant to the frequency location of the LF and HF spectral peaks is also provided (each circle has coordinates (*f*_*q*_, $\sigma_{q}^{2}$ ), where *q* represents the LF or HF band). While the tendency of the LF power is towards an increase moving from REST to TILT (*p* = 0.068), the HF power significantly decreases (*p* = 0.002). Furthermore, the assessment of the significance of the difference between power values integrated in LF and HF bands in a given condition revealed that the latter ones are predominant (*p* < 0.001) during the supine rest (Figure 3A, $\sigma_{LF}^{2}$ vs. $\sigma_{HF}^{2}$ ). This finding may reflect the fact that HF oscillations of AC can be heavily affected by several respiration-related mechanisms, including (i) the direct mechanical effect of intrathoracic pressure oscillations on the arterial wall stretch, and (ii) the effect of HF oscillations in AC modulators such as heart rate and ABP, with former bringing information about the mechanisms of respiratory sinus arrhythmia (RSA) (Elstad and Walløe, 2015; Švec and Javorka, 2021; Wiszt and Javorka, 2022). The predominance of HF withdraws with tilt due to a slight increase of LF power (*p* = 0.068) and a significant decrease of HF power (*p* = 0.002), as depicted in Figure 3B. An increase of magnitude of LF oscillations can reflect the sympathetically-driven vasomotion as a result of its baroreflex-mediated activation associated with orthostasis (Cooper and Hainsworth, 2001; Nardone et al., 2018; Czippelova et al., 2019). Conversely, a decrease of magnitude of HF oscillations could be attributed to the parasympathetic inhibition during orthostasis reflected by decreased RSA magnitude (Berntson et al., 1994; Javorka et al., 2018). Quantifying the effects of potential drivers of AC oscillations, such as changes of heart rate and TPR, could improve our understanding of the observed changes in AC variability (Czippelova et al., 2019; Švec et al., 2021).

**FIGURE 4:**
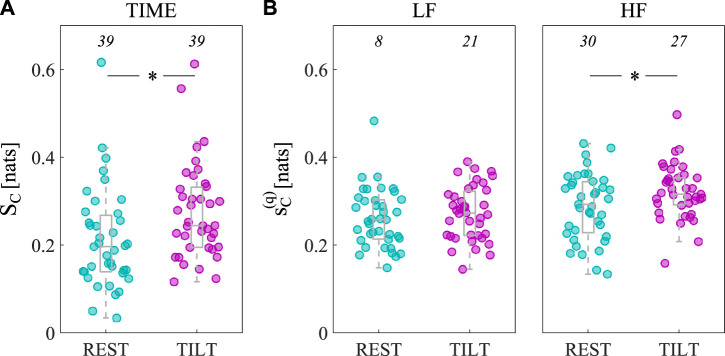
Assessment of arterial compliance self-predictability in the time and frequency domain. Measures of LSP integrated in time (*S*_*C*_, **(A)**, LF ( $s_{C}^{\left(LF\right)}$ , **(B)**, left) and HF ( $s_{C}^{\left(HF\right)}$ , **(B)**, right) bands of the spectrum, in REST (left boxplots, cyan circles) and TILT (right boxplots, magenta circles) conditions. The total number of subjects is 39. Numbers in each plot indicate statistically significant LSP values in REST (left) and TILT (right) according to surrogate and bootstrap data analyses. Statistically significant differences assessed by the Wilcoxon signed-rank test for paired data: ∗, *p* < 0.05 REST vs. TILT.

In Figure 4, the spectral representation of the LSP is shown in terms of boxplot distributions of the integrated measure over all frequencies (*S*_*C*_, Figure 4A), as well as in the LF ( $s_{C}^{\left(LF\right)}$ , Figure 4B, left) and HF ( $s_{C}^{\left(HF\right)}$ , Figure 4B, right) bands, computed in the REST (left boxplots, cyan circles) and TILT (right boxplots, magenta circles) conditions. The significant increase of the time domain LSP moving from REST to TILT (*p* = 0.002) is confirmed only in the HF band of the spectrum (*p* = 0.007). This suggests that the overall increase of regularity of the process cannot be generalized to the whole frequency content of arterial compliance, but is rather confined to the HF band of the spectrum and may have different origins.

One of them is related to the mathematical nature of the LSP measure $s_{C}^{\left(F\right)}\left(f\right)$ (*F* represents the LF or HF band), whose spectral profile is given by the sum of the frequency-independent term *S*_*C*_, and the zero-mean term ${\bar{s}}_{C}^{\left(F\right)}\left(f\right)$ showing a peak in band *F*. Potential tilt-induced significant increases of *S*_*C*_ are thus frequency-independent and distributed uniformly along the whole frequency axis. Then, the spectral LSP $s_{C}^{\left(F\right)}\left(f\right)$ is influenced by this increase even in the case when there is no significant change in fluctuations of ${\bar{s}}_{C}^{\left(F\right)}\left(f\right)$ ; this influence has major weight in the larger spectral band due to the higher number of integrated frequency bins (i.e., the HF band), and may be thus responsible for the observed change of self-predictability in this band.

From a physiological point of view, the degree of complexity of arterial compliance could be a result of the combined effects of external influences modulating its dynamic activity and operating over different temporal scales, such as direct mechanical or neural influences arising from central oscillators (respiratory and vasomotor oscillators), feedback loops (e.g., baroreceptive closed-loop circuit), and complex physiologic mechanisms adjusting TPR, ABP and heart rate.

At first sight, the unaltered regularity of AC in the LF band can be attributed to a hidden tilt-induced sympathetic activation, due to the high degree of co-ordination and synchronicity of several simultaneous control mechanisms regulating AC in both the resting state and tilt conditions (e.g., heart rate, blood pressure and TPR). However, bootstrap data analysis yielded opposite results, since we found that the significance of AC self-predictability in the LF band increased with tilt (from 8/39, i.e., 20.51%, in the supine rest to 21/39, i.e., 53.85%, in tilt), as depicted in Figure 4B (left). This finding is of great importance and confirms the slight activation of sympathetic vasomotor control observed for the power spectral density of compliance (Cooper and Hainsworth, 2001; Nardone et al., 2018; Czippelova et al., 2019), besides possibly reflecting an increased amplitude of LF oscillations in external modulators such as ABP or TPR (Cooke et al., 1999; Elstad et al., 2011). Noteworthy, the augmented number of significant spectral measures in this band can be explained by considering the subject-specific frequency profiles of $s_{C}^{\left(LF\right)}\left(f\right)$ , which are likely to show more prominent peaks in LF during tilt, in accordance with wider fluctuations of ${\bar{s}}_{C}^{\left(LF\right)}\left(f\right)$ and in spite of an overall frequency-independent increase of *S*_*C*_ (as shown in Figure 4A), i.e., the threshold for assessing significance in bootstrap data analysis (Sect. 2.3.2).

The tilt-induced physiologic responses resulting in decreased respiratory rates and increased tidal volumes (Brown et al., 1993; Porta et al., 2000; Javorka et al., 2018), as well as in a slight diminished complexity of the respiration signal (Valente et al., 2017), may be responsible for the increase of regularity of AC in the HF band. Indeed, the increased mechanical influences on arterial vessels due to an augmented tidal volume are likely to produce an augmented coupling between arterial stiffness and respiration, reflected by an increase of AC self-predictability in the HF band. Moreover, an increased HF-related regularity in AC could be attributed also to the effect of increased magnitude of ABP variability in this band (Cooke et al., 1999), probably resulting from the tilt-induced suppression of buffering effect of RSA on ABP variability at the respiratory frequency (Cooke et al., 1999; Elstad et al., 2001). Noteworthy, the latter findings highlight one important limitation of the LSP measure, which is its formulation in absence of a multivariate context taking into account potential oscillatory external drivers of AC variability, such as ABP and respiration. A spectral measure of autonomous dynamics defined as Granger Autonomy has been proposed recently, which is an extension of the LSP to the bivariate case and takes into account potential confounding mechanisms deriving from external sources (Sparacino et al., 2023). It is worth noting that the significance of HF self-predictability decreases from 30/39 (76.92%) in the supine rest to 27/39 (69.23%) during tilt, as depicted in Figure 4B (right). One more time, this result could be interpreted by looking at the spectral profiles of $s_{C}^{\left(HF\right)}\left(f\right)$ : while the increase of self-predictability in HF may be associated to the frequency-independent increase of the term *S*_*C*_, the tilt-induced diminished significance of the spectral LSP in the same band could be the result of less prominent peaks due to dampened fluctuations of ${\bar{s}}_{C}^{\left(HF\right)}\left(f\right)$ . Again, if combined with the increase of significance of LF regularity, this result confirms a parasympathetic withdrawal related to heart rate control and suggests the importance of LF fluctuations when the process has to cope with the physiological perturbations due to the orthostatic challenge.

The application of the proposed approach to arterial compliance data has demonstrated the significance of computing frequency-specific self-predictability measures in the case of physiological variables rich of oscillatory components with different frequencies and shape, suggesting that the overall changes of self-predictability in the time domain may be confined to specific bands of the spectrum. Moreover, investigating the spectral self dynamics of physiological processes may have a great impact in understanding their role in multivariate contexts.

## 5 Conclusion

In this study, we proposed a novel spectral measure to characterize the self-predictability of linear Gaussian physiologic processes in the frequency domain. The spectral function was defined in such a way to be related to the peaks of the PSD, which here was represented as the sum of different spectral components with specific power and frequency through the well-established method of spectral decomposition (Baselli et al., 1997; Pernice et al., 2021). Yet, in spite of being a simple mathematical tool derived from the TF of the process and reflecting its spectral dynamics, the proposed spectral LSP has a clear meaning in the field of information theory as it is related to the well-known concept of information storage. Moreover, due to the logarithmic transformation of the TF, the effect of outliers is minimized and changes in the auto-dependencies are amplified vertically, thus allowing to differentiate their behavior in practical computations with respect to other non-logarithmic measures (e.g., the variance of the poles of the power spectrum). Noteworthy, being particularly useful for the analysis of dynamic processes which are rich of oscillatory content, the proposed method has the potential to infer physiological mechanisms which can be hidden in time domain due to the mixing with other spectral effects, as well as to more precisely quantify the spectral information content associated to specific oscillations of the process. These capabilities are demonstrated first in a theoretical example of a simulated AR process, and then in the application to short-term beat-to-beat variability of noninvasive estimates of arterial compliance acquired in young healthy subjects in the supine resting position and during head-up tilt (Švec et al., 2021; Švec and Javorka, 2021). The observed shift of oscillatory content of compliance towards low frequencies is associated to an increase of the significance of linear self-predictability in LF, as confirmed by the spectral decomposition of the LSP, and probably due to the tilt-induced augmented sympathetic control and vagal withdrawal.

Remarkably, the proposed framework can be generalized to any physiologic variable exhibiting oscillatory activity, e.g., to cardiovascular, respiratory, cerebrovascular and neural data. We remark indeed the generality of the linear parametric representation of information-based measures such as the LSP, that naturally lends itself to the frequency-specific analysis of oscillatory processes.

The present study has two significant methodological limitations. First, being based on univariate metrics, it does not take into account that linear predictability of the investigated process may change when the system is part of a network of physiological interactions. Hence, the interpretation of *S*_*Y*_ as a measure of self-predictability may be confounded by the fact that it also includes dynamical influences which stem not only from the investigated target system, but also from other potentially interconnected source systems (Wibral et al., 2014). Therefore, future studies should be focused on the use of bivariate or even multivariate parametric models, analyzed in the time and/or spectral domains, to investigate the role of other systemic variables in the potential generation of the target oscillations. As regards our application to arterial compliance, the influence of putative drivers controlled by autonomic nervous system (blood pressure, heart rate), together with the mechanical effects of intrathoracic pressure changes during respiration and the role of other factors that contribute to compliance beat-to-beat variability (e.g., the time constant *τ* or TPR), should be investigated to clarify their effects on AC variability. A secondary but still considerable weakness of this study is related to the methodology. Indeed, it is worth noting that the proposed spectral LSP measure could probably be affected by the broadband aperiodic $\frac{1}{f}$ component, previously investigated in some very interesting works (Yamamoto and Hughson, 1991; Wen and Liu, 2016; Donoghue et al., 2020). In the short-term setting like the one considered in our analysis, this effect is likely to determine information stored at very low frequencies, meaning that it could result in spectral components identified by poles of the TF which are very close to zero, describing trends in the observed time series that look aperiodic. This is a very interesting aspect which deserves specific thorough investigation in further studies on the topic.

## Data Availability

The raw data supporting the conclusion of this article will be made available by the authors, without undue reservation.
